# Cellulolytic RoboLector – towards an automated high-throughput screening platform for recombinant cellulase expression

**DOI:** 10.1186/s13036-016-0043-2

**Published:** 2017-01-07

**Authors:** Martina Mühlmann, Martin Kunze, Joaquim Ribeiro, Bertram Geinitz, Christian Lehmann, Ulrich Schwaneberg, Ulrich Commandeur, Jochen Büchs

**Affiliations:** 1 AVT-Chair for Biochemical Engineering, RWTH Aachen University, Worringerweg 1, 52074 Aachen, Germany; 2 Chair for Biotechnology, RWTH Aachen University, Worringerweg 1, 52074 Aachen, Germany; 3 Chair for Molecular Biotechnology, RWTH Aachen University, Worringerweg 1, 52074 Aachen, Germany

**Keywords:** High-throughput screening, Automation, On-line monitoring, Cellulase, Microtiter plate

## Abstract

**Background:**

Cellulases are key player in the hydrolyzation of cellulose. Unfortunately, this reaction is slow and a bottleneck in the process chain from biomass to intermediates and biofuels due to low activities of the enzymes. To overcome this draw back, a lot of effort is put into the area of protein engineering, to modify these enzymes by directed evolution or rational design. Huge clone libraries are constructed and have to be screened for improved variants. High-throughput screening is the method of choice to tackle this experimental effort, but up to now only a few process steps are adapted to automated platforms and little attention has been turned to the reproducibility of clone rankings.

**Results:**

In this study, an extended robotic platform is presented to conduct automated high-throughput screenings of clone libraries including preculture synchronization and biomass specific induction. Automated upstream, downstream and analytical process steps are described and evaluated using *E. coli* and *K. lactis* as model organisms. Conventional protocols for media preparation, cell lysis, Azo-CMC assay and PAHBAH assay are successfully adapted to automatable high-throughput protocols. Finally, a recombinant *E. coli* celA2 clone library was screened and a reliable clone ranking could be realized.

**Conclusion:**

The RoboLector device is a suitable platform to perform all process steps of an automated high-throughput clone library screening for improved cellulases. On-line biomass growth measurement controlling liquid handling actions enables fair comparison of clone variants.

**Electronic supplementary material:**

The online version of this article (doi:10.1186/s13036-016-0043-2) contains supplementary material, which is available to authorized users.

## Background

Since fossil resources are limited, many current research projects are investigating the utilization of renewable resources to ensure the sustainable production of platform chemicals and biofuels [1]. Most of these approaches have focused on producing alcohols from starch-containing plant material which competes with the food supply chain. Moreover, these approaches waste most of the plant biomass. New research is focusing on utilizing ligno-cellulose as the prime raw material for biofuel production [2–4] and constructing new biocatalysts for this purpose [5].

As the cellulose de-polymerization performed by different kinds of cellulases is the rate-limiting step for the whole hydrolysis [6, 7], efficient cellulases have to be identified and characterized regarding their performance. Mutagenesis procedures are a common way to modify existing enzymes in order to improve their properties. Typically, such modifications end up in huge libraries of enzyme variants which need to be further investigated [8]. For this task an efficient screening process is essential [6, 9]. To handle this immense workload, a high-throughput screening (HTS) system is necessary, which combines a miniaturized mode of operation with an advanced automation concept [10].

Automated high-throughput (HT) concepts for single steps such as measurement of optical density, pH, metabolites [11], protein purification [12–14] and different enzyme activity assays [11, 15, 16] have been reported.

More advanced HT concepts combine single methods to a semi or even fully automated operation. Dörr et al. [17] recently presented a robotic platform for HT enzyme library screening. It enables automated colony picking, at-line monitoring of biomass growth, induction, subsequent enzyme purification steps and biochemical assay. However, this platform lacks in an on-line monitoring tool to follow the biomass growth without interruption of shaking. In particular, constant oxygen transfer rates are necessary for reproducible cultivations. Huber et al. [18] combined the BioLector technology [19] and a robotic liquid handling system to overcome this issue. A similar set-up was later used by Hemmerich et al. [20] who performed an on-line monitored fed-batch clone screening of *Pichia pastoris*. However, the automation was restricted to the feeding and harvest procedure. Both, the preculture and the subsequent enzyme activity assay was conducted manually. A more complex clone screening was carried out with *C. glutamicum* by Unthan et al. [21]. They integrated an automated biomass triggered sampling with subsequent separation of supernatant by centrifugation and a following freezing step. An independent assay was used for the analysis of samples. Unfortunately, no conditioning of the preculture was attempted since these authors inoculated directly from cryo cultures into the main culture. Additionally, there was no further information about the generation of replicates. It’s important to distinguish technical replicates from biological replicates. A small standard error in technical replicates doesn’t reflect the reliability of the whole experimental procedure. It only gives information about the reliability of the last measurement step. Moreover, there is a difference in using one microtiter plate (MTP) for producing replicates at the same time and the repetition of a whole experiment in many plates at different times. The big challenge is the comparability of screening results amongst different plates. Clone screening commonly starts from 96well cryo cultures whose biomass concentrations are not individually determined or adjusted. Without the application of on-line measurement tools, this typically leads to a usually non-recognized difference in onset of growth, when comparing different variants. Thereupon, especially microorganisms which have to be induced undergo non-comparable enzyme expression [22, 23].

In this study, an automated and reliable HT system is presented which is able to realize all necessary steps for a complete screening of cellulolytic enzymes. The applied methods focus especially on preculture synchronization and controlled induction of clone libraries which is a prerequisite to obtain a fair comparison of all screened variants. Based on the RoboLector system described before [18], an extended version was constructed to realize more complex operations. Furthermore, existing methods for protein purification and cellulase characterization were adapted to and optimized for miniaturized HT operation with a special focus on automated handling. In several application examples, the potential of the extended cellulolytic RoboLector system is demonstrated. Single steps were evaluated using different *E. coli* and *K. lactis* strains*.* While *E. coli* usually cannot secrete recombinant proteins, *K. lactis* possesses a secreting system. This leads to a more complex downstream process for *E. coli*. In this study, 46 mutants of *E. coli* expressing celA2 were used for the final evaluation of the screening procedure. A *K. lactis* mutant library screening was not part of this study.

## Methods

### Microorganisms

The applied microorganisms with their respective vectors for recombinant protein expression as well as their selection markers are specified in Table 1.

**Table 1:** Applied microorganisms for the expression of recombinant proteins

Target protein	Organism	Strain	Vector	Selection marker	Inducer
FbFP	*E. coli*	BL21 (DE3)	pRhotHi-2	Kanamycin	Lactose
cel8H-mCherry	*E. coli*	BL21 (DE3) pLys	pET-22b(+)	Ampicillin	Lactose
celA2	*E. coli*	BL21 (DE3)	pET-28a(+)	Kanamycin	IPTG
cel5A	*K. lactis*	GG799	pKlac1	-	Galactose

The *Escherichia coli* (*E. coli*) clone library for the expression of celA2 variants was created by directed evolution applying a simultaneous site-saturation mutagenesis at positions 288, 299 and 300. Active variants where picked from LB agar plates supplemented with 0.125% (w/v) Azo-CMC. Active transformants which showed halos were picked. Similar libraries were constructed and investigated by Lehmann et al. [24].

### Media & cultivation

For *E. coli* expressing FbFP and cel8H-mCherry terrific broth (TB) medium [25] consisting of 12 g L^−1^ tryptone, 24 g L^−1^ yeast extract, 12.54 g L^−1^ K_2_HPO_4_, 2.31 g L^−1^ KH_2_PO_4_, and 5 g L^−1^ glycerol (all ingredients from Roth, Germany) dissolved in water was used for preculture. The pH value was 7.2 ± 0.2 without adjustment. If not stated differently, 10 mL of TB medium in a 250 ml shake flask were inoculated with 50 μL from a cryoculture for *E. coli* pre-cultivation, and cultures were grown for 8 h at 350 rpm (shaking diameter 50 mm) and 37 °C. For main culture, OvernightExpress^TM^ (OnEx) medium was used for auto-induction of the FbFP strain. Besides this, the cultivation conditions were identical to the preculture. For expression of cel8H-mCherry, a modified TB medium was used containing varied amounts of glucose and lactose in addition to 5 g L^−1^ glycerol (see Table 2). The cultivation was performed within a 96well MTP (Greiner Bio-One GmbH, Frickenhausen, Germany) with 200 μL filling volume and a shaking frequency of 1000 rpm (shaking diameter 3 mm) at 37 °C. The plates were sealed with gas-permeable seals (AB-0718, Thermo Scientific, Dreieich, Germany).

**Table 2:** Varied composition of auto-induction media and their abbreviations for the recombinant expression of the cel8H-mcCherry fusion protein in *E. coli*

Abbreviation	Glycerol [g L^−1^]	Glucose [g L^−1^]	Lactose [g L^−1^]
5052	5	0.5	2
512	5	1	2
522	5	2	2
532	5	3	2
516	5	1	6
5110	5	1	10

For the pre- and main cultivation of *E. coli* expressing celA2 a modified Wilms and Reuss medium (henceforth referred as Wilms-MOPS medium) was used [26]. It consists of 5 g L^−1^ (NH_4_)_2_SO_4_, 0.5 g L^−1^ NH_4_Cl, 3.0 g L^−1^ K_2_HPO_4_, 2 g L^−1^ Na_2_SO_4_, 0.5 g L^−1^ MgSO_4_ · 7H_2_O, 0.01 g L^−1^ thiamine hydrochloride, 41.85 g L^−1^ 3-(N-morpholino)-propanesulfonic acid (MOPS, 0.2 M), 15 g L^−1^ glucose and 1 mL L^−1^ trace element solution. This trace element solution consists of 1.98 g L^−1^ CaCl_2_ · 2H_2_O, 0.54 g L^−1^ CoCl_2_ · 6H_2_O, 0.48 g L^−1^ CuSO_4_ · 5H_2_O, 41.76 g L^−1^ FeCl_3_ · 6H_2_O, 0.3 g L^−1^ MnSO_4_ · H_2_O, 0.54 g L^−1^ ZnSO_4_ · 7H_2_O, 33.39 g L^−1^ Na_2_EDTA (Titriplex III). The pH was adjusted with 5 M NaOH to a value of 7. Pre- and main culture were performed within a 96well MTP (Greiner Bio-One GmbH, Frickenhausen, Germany) and sealed with xPierce™ film (Z722529, Sigma-Aldrich, Munich, Germany). The expression of celA2 in *E. coli* was individually induced after exceeding the scattered light value of 10 a.u. by automated adding of IPTG, resulting in a final concentration of 0.1 mM.

Depending on the clone’s resistance 50 μg mL^−1^ kanamycin or 100 μg mL^−1^ ampicillin were added to all the media from a 1000-fold concentrated stock solution.

For *Kluyveromyces lactis* (*K. lactis*) precultures yeast extract peptone (YP) medium was used, consisting of 10 g L^−1^ yeast extract, 20 g L^−1^ tryptone (both Carl Roth, Karlsruhe, Germany) and 10 g L^−1^ glucose [27]. 10 mL of this medium were inoculated with 50 μL from a *K. lactis* cryoculture, and cultures were grown for 12 h at 350 rpm (shaking diameter 50 mm) and 30 °C in a 250 ml shake flask. The main cultures were performed in YP medium containing 10–100 g L^−1^ galactose instead of glucose. Thereby, galactose served as carbon source and inducer for recombinant protein expression. The cultivation was conducted within so-called Flower Plates (MTP-48-BOH, Lot. 1202, m2p - labs, Germany), equipped with optodes for on-line monitoring of the dissolved oxygen tension (DOT). Flower Plates were sealed manually with gas-permeable seals (AB-0718, Thermo Scientific, Dreieich, Germany).

All MTP cultivations were performed applying the BioLector technique [19]. For this purpose, the commercial device from m2p-labs (Beasweiler, Germany) was used. Microbial growth and formation of fluorescent proteins were on-line monitored. Wavelengths and gain factors for all optical signals are listed in Table 3. For scattered light and fluorescence measurement the initial light intensity (I_0_), which is mainly attributed to such factors as the media background or the type of the MTP, was subtracted from the original measured data (I-I_0_).

**Table 3:** Optical signals and applied setup for BioLector on-line monitoring

Optical signal	λ_ex_ [nm]	λ_em_ [nm]	Gain
Biomass (scattered light)	620	-	20
DOT	520	600	60
mCherry fluorescence	580	610	60
FbFP fluorescence	450	492	60

### Offline analysis

OD_600_ was determined via a Genesys 20 photometer (Thermo Scientific, Dreieich, Germany) in 1.5 mL micro cuvettes (PS, Plastibrand, Roth, Karlsruhe, Germany). For values higher than 0.5 the samples were appropriately diluted with 0.9% [m/v] NaCl solution.

For FbFP fluorescence quantification, 100 μl of the respective sample were transferred to a cavity of a 96well MTP (Greiner Bio-One GmbH, Frickenhausen, Germany). The measurement was done applying the BioLector system with parameters listed in Table 3. Each presented value is the average of at least 10 consecutive measuring values.

SDS-PAGE *-* After measuring the OD_600_ of the culture, samples were centrifuged at 18,000 g for 10 min. Subsequently, the supernatant was removed, and the pellet was re-suspended in water to an OD_600_ of 25. To 40 μL of this suspension, 140 μL of twofold concentrated sample buffer (E-PAGE Loading Buffer, Invitrogen, Germany) and 20 μL of 1 M dithiothreitol (AppliChem GmbH, Darmstadt, Germany) were added before shaking the mixture for 10 min at 1000 rpm and 70 °C in a thermo shaker (MKR 10, HLC Biotech, Bovenden, Germany). For analysis, the SDS-PAGE device (Invitrogen, Germany) was equipped simultaneously with up to two gels (4–12% Bis-Tris, Invitrogen, Germany). The transferred volumes were 20 μL for the prepared samples and 15 μL for the protein marker (RotiVR -Mark Standard, Roth, Germany). The process was operated under the suggested standard settings of the manufacturer (running time 35 min, maximum current 200 V, and maximum power 0.25 W). The gels were stained in Simply Blue Staining solution (Invitrogen, Germany) at 37 °C and shaken at 60 rpm overnight and were subsequently washed in water at the same temperature and shaking conditions for 2 h.

4-MUC assay – The endoglucanase activity of the recombinant protein celA2 was measured manually with the already established fluorescence-based 4-methylumbelliferyl-β-D-cellobioside (4-MUC) assay [24]. Some adaptions were made. A 50× 4-MUC stock solution (0.5 mM) was prepared by dissolving the reagent in potassium phosphate buffer (0.1 M, pH 6.5) and stored at 4 °C. 40 μL of disrupted cells and 90 μL of a diluted 4-MUC solution (1.67×) were transferred separately into a 96well MTP (flat-bottom, black/clear, BD Falcon, USA), sealed with a clear sealing tape (EN77.1, Roth, Germany) and preheated to 30 °C. After the pre-incubation, 60 μL of the 4-MUC solution were mixed with the disrupted cells. Subsequently, the reaction mixture was incubated at 30 °C for 20 min in a Synergy 4 Microplate Reader (BioTek, Winooski, VT, USA). The increase in fluorescence was measured at Ex/Em = 365/455 nm in intervals of 1 min. For evaluation, a calibration curve, fluorescence versus 4-methylumbelliferone concentration (0.3–300 μM), was recorded in the same phosphate buffer and a final volume of 100 μL. Specified activity values for all measured clone variants refer to the volumetric activity within the culture broth.

### RoboLector system

**Fig. 1:**
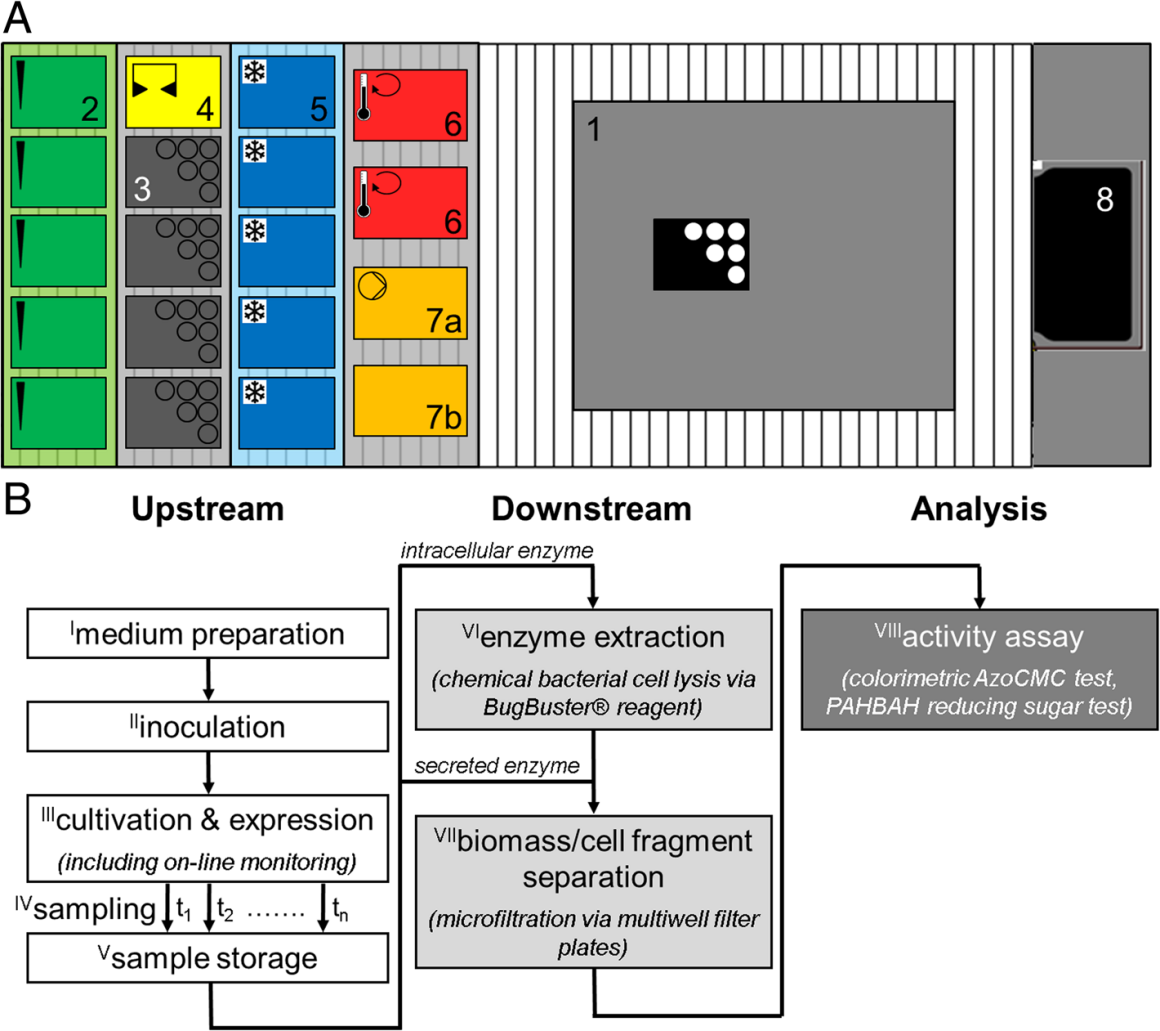
Automated HTS of recombinant cellulase expression applying the RoboLector platform. **a** Layout of the automated HTS RoboLector platform: *1*) Customized BioLector on-line monitoring device; *2*) Pipetting tip carrier for up to 5× 96 tips; *3*) Carrier with 4 MTP storage positons; *4*) Gripping tool parking position; *5*) Cooling carrier for storage of up to 5 MTPs containing samples and/or chemicals; *6*) Heater shakers (2×) for MTPs; *7a*) Vacuum filtration module for one filter MTP with vacuum chamber, *7b*) Lid parking position; and *8*) Waste bag. **b** Schematic protocol for an automated HTS procedure for recombinant cellulase expression with processing steps I-VIII

The applied automated HTS platform RoboLector is identical to the system described by Huber et al. (2009). For this work, it was extended by the following components which are commercially available from Hamilton Robotics GmbH (Martinsried, Germany): cooling carrier (PLT_CAR_L5C), MTP heater shakers (HHS), on-deck vacuum station (BVS). The extended setup is illustrated in the layout scheme in Fig. 1a. The cooling carrier was operated with a controllable recirculation cooler (FL300, Julabo, Seelbach, Germany) located under the robotic platform and connected via flexible tubes. It allows temperature set points from −20 °C to 40 °C. To keep the tubes from ice coating, set temperatures lower than −5 °C were avoided for long-term experiments. Due to heat transfer phenomena, set temperatures and final temperatures in MTP wells differ from each other. The respective relationship was identified by experiments. For 96well deep well plates (DWP, 2.2 mL, sterile, square wells, Corning/Axygen Scientific) it is described by Eq. 1.

1 $$ {T}_{well}=0.75\cdot {T}_{set}+6.50{}^{\circ}C $$

Similar experiments were performed for the heater shakers, too. The respective relationship for 96well DWPs is described by Eq. 2. The maximum set temperature was 110 °C. Well temperatures were determined by a CheckTemp 1 precision thermometer (G0021, Roth, Karlsruhe, Germany).

2 $$ {T}_{well}=0.78\cdot {T}_{set}+6.45{}^{\circ}C $$

The vacuum station was operated by a vacuum pump (ME 4C VARIO, Vacuubrand, Wertheim, Germany) equipped with pressure sensor and control unit. All devices are controlled by the VENUS three software of the pipetting robot (Microlab STAR, Hamilton Robotics, Martinsried, Germany).

### HT biomass separation

For the HT separation of whole cells or cell fragments, microfiltration was performed applying the on-deck vacuum station of the RoboLector (Fig. 1a, no. 7a-b) with AcroPrep^TM^ 96 Filter Plates (3.0 μm Glass Fiber/0.2 μm Bio-Inert membrane, modified hydrophilic nylon, 1 mL, part no. 5053, Pall Life Sciences, Ann Arbor, MI, USA). For the filtration an underpressure of 0.5–0.6 bar was applied. Previous experiments revealed that a filtration time of 30 min is sufficient for the complete filtration of cultivation samples with volumes up to 600 μL and OD_600_ values up to 20. For all HT filtration experiments, full plate occupancy was ensured for a homogeneous vacuum distribution.

### Enzyme extraction

For *E. coli* cell lysis and protein extraction, the commercial kit BugBuster® 10× Protein Extraction Reagent (70921, Merck-Millipore, Darmstadt, Germany) was used. It is a detergent based mixture with a small amount of lysozyme. For the standard protocol, samples were filled in 1.5 mL tubes, biomass was separated via centrifugation for 5 min at 18,000 g (centrifuge 1–15 K, rotor 12024-H, Sigma), and the supernatant removed with a pipette. For re-suspension, the 10× Protein Extraction Reagent was diluted in acetate buffer (to 1×) and a volume equal to the original sample volume was added to the pellet. After 30 min of incubation at 900 rpm and room temperature, samples were centrifuged again for 5 min at 18,000 g. The resulting supernatant containing the target protein was stored at 4 °C. It must be noticed that this protocol is not in accordance with the manufacturer’s guidelines since it was optimized for internal use. For the HT protocol, cultivation samples were given directly to a multiwell filter plate for microfiltration. For protein extraction, 11 μL of 10× Protein Extraction Reagent was added to each 100 μL sample volume. After 30 min of incubation at 900 rpm and room temperature, the filter plate was automatically transported by the gripper to the on-deck vacuum station of the RoboLector and filtration performed as described for microfiltrations. The filtrate was collected in a 96well DWP and, if necessary, stored at 4 °C.

### Azo-CMC assay

Azo-CMC is a commercially available substrate for endo-1,4-β-glucanases (Megazyme, Bray, Ireland). All working solutions were prepared as given by the manufacturer’s instructions. Only the acetate buffer’s pH was adjusted to 4.8 instead of 4.6. For the standard protocol of the assay, 0.5 mL enzyme sample were added to 0.5 mL Azo-CMC solution in a 5 mL tube. Both, enzyme and substrate solution were pre-heated to the incubation temperature of 45 °C. Incubation was done for 10 min at 900 rpm, before adding 2.5 mL precipitant solution. After thorough manual mixing, the sample was incubated at RT for 10 min. Precipitated non-hydrolyzed substrate was separated by centrifugation at 18,000 g for 10 min. For measurement, 100 μL of supernatant were transferred to a flat-bottom MTP (9293.1, Roth, Germany) and absorbance was determined at 590 nm in a Synergy 4 Microplate Reader (BioTek, Winooski, VT, USA). For the HT protocol, 0.2 mL of the enzyme sample were added to 0.2 mL Azo-CMC solution in a 96well DWP. Both, the MTP containing the samples and the MTP with the substrate solution were pre-heated on the heater shakers (Fig. 1a, no.6) for 15 min at 45 °C. After the incubation for 10 or 20 min, respectively, at 900 rpm, 1 mL precipitant solution was added. Mixing was done by threefold repeatedly aspirating and ejecting the solution with the pipetting robot. After 10 min incubation at RT, 300 μL of the solution were transferred to an AcroPrep^TM^ 96 Filter Plate (0.2 μm Bio-Inert membrane, modified hydrophilic nylon, 350 μL, part no. 5042, Pall Life Sciences, Ann Arbor, MI, USA) placed on the vacuum station of the RoboLector. For complete filtration, an underpressure of 0.5–0.6 bar was applied for 15 min. To ensure homogeneous vacuum distribution, filtrations were performed with full plate occupancy. For measurement, 100 μL of filtrate were transferred from the receiver plate to a flat-bottom MTP and absorbance was determined at 590 nm in a Synergy 4 Microplate Reader (BioTek, Winooski, VT, USA). To determine calibration curves, a commercially available endo-1,4-β-D-glucanase (EGII) from *Trichoderma longibrachiatum* was used (E-CELTR, Megazyme, Bray, Ireland). By dilution in 0.1 M acetate buffer, measuring solutions were prepared with cellulase activities of 100–1000 mU ml^−1^.

### CMC hydrolysis

For CMC hydrolysis under HT conditions, 500 μL of enzyme samples were given to 500 μL of a pre-heated solution of 10 g L^−1^ CMC in 0.1 M acetate buffer in a 96well DWP on a heater shaker of the RoboLector system. After incubation at 900 rpm and 45 °C, samples were either transferred to a DWP plate on the cooling carrier and stored at 4 °C or directly analyzed via PAHBAH test.

### PAHBAH test

For the performance of the PAHBAH reaction, the respective working solution was prepared directly before performing the test by mixing one part of reagent A and nine of reagent B. The working reagent remains stable for only a few hours. For reagent A, 5 g 4-hydroxybenzoic acid hydrazide, 30 mL H_2_O (dist.), and 5 mL HCl (37%) are mixed and filled up to 100 mL with H_2_O (dist.). For reagent B, 12.5 g trisodium citrate, 1.1 g CaCl_2_, and 20 g NaOH are dissolved in 500 mL H_2_O (dist.) and filled up to 1 L with H_2_O (dist.). Both reagents are stable for approximately 3–4 weeks. For the standard protocol, 75 μL sample were mixed with 150 μL working reagent in a 2 mL tube and incubated for 10 min at 900 rpm and 100 °C on a thermo mixer (MHR23, HLC Biotech, Bovenden, Germany). After cooling to room temperature, 100 μL were transferred to a flat-bottom MTP (9293.1, Roth, Germany) and absorbance was measured at 410 nm (Synergy 4 Microplate Reader, BioTek, Winooski, VT, USA). For calibration, reference standards with 0–0.5 g L^−1^ glucose in 0.1 M acetate buffer were used. For the HT protocol, 75 μL sample were given to 150 μL pre-heated working reagent in a 96well DWP and incubated for 10 min at 900 rpm and 85 °C on a heater shaker of the RoboLector system. Afterwards, 100 μL were transferred to a flat-bottom MTP and absorbance was measured at 410 nm. For calibration, reference standards containing 10 g L^−1^ CMC and 0–0.5 g L^−1^ glucose in 0.1 M acetate buffer, as well as pure buffer were used. The absorbance difference for a buffer with 10 g L^−1^ CMC and pure buffer was subtracted from each measuring value.

## Results & discussion

### Extended RoboLector HTS platform

To meet the demands for an automated HTS of cellulase expression, the original RoboLector system [18] had to be extended. Figure 1a shows the layout of the robotic platform equipped with its different components. The conventional RoboLector system contained the custom-built BioLector on-line monitoring system (1), a carrier for pipetting tip storage (2), a carrier for the storage of different MTPs (3) with an additional position to park the iSwap gripping tool (4), and a waste bag for used tips and MTPs (8). With this setup a sufficient upstream process including media preparation, inoculation, cultivation (including on-line monitoring) as well as automated sampling is possible. But, it lacks possibilities for further sample processing like storage, purification and analysis. Therefore, additional components had to be added to the robotic platform. For tempering tasks, two further tools were integrated. A cooling carrier with five MTP positions (5) connected to a controllable recirculation cooler allows storage of samples and chemicals at low temperatures. With two MTP shakers (6), heat treatment at temperatures up to 110 °C can be performed. Furthermore, the RoboLector system was extended by a vacuum filtration module for micro- and ultrafiltration steps in special multiwell filter plates. The filtration module consists of the vacuum chamber (7a), also containing the filtrate receiver plate, and a parking position for the chamber’s lid (7b). The components 2–8 are commercially available from Hamilton Robotics GmbH (Martinsried, Germany).

Figure 1b shows the schematic protocol for automated HTS experiments with the necessary process steps for cellulase expression, purification and quantification. Depending on the experiment’s needs, single steps can, of course, be skipped. The screening process can be classified into three major phases: upstream, downstream, and analysis. The upstream part starts with the preparation of the cultivation medium direct in the cultivation MTP from up to 15 supplied stock solutions stored in DWPs on the cooling carrier (step I). Thereby, the combination of automated medium preparation and cultivation in MTPs allowed to compare different media or to investigate the influence of one or more medium components in short time. Subsequently, the medium is inoculated from a prepared MTP containing the preculture which is shaken on a MTP shaker to ensure a homogeneous suspension (step II). The inoculated cultures are then cultivated under defined conditions in the BioLector system, accompanied by optical on-line monitoring of relevant fermentation parameters such as biomass and product formation, DOT and pH (step III, [19, 28]). If necessary, chemicals can be added to the cultures by the pipetting robot at pre-defined times, e.g. inducing chemical compounds to start product formation. At pre-defined time points, samples can fully automatically be taken (step IV) and stored in MTPs on the cooling carrier for further processing (step V). Both, the adding of substances as well as taking samples cause only short interruptions of the cultivation process so that no negative influences are observed.

The upstream phase ends when the cultivation is finished and final samples are taken and stored. The subsequent downstream processing is focused in this work on the elimination of cell material from the cultivation samples. The fact that the target enzyme is either produced intracellular or secreted to the medium dictates which route is taken to gain a cell free cellulase solution. For extracellular enzymes a microfiltration step applying multiwell filter plates with a maximum pore size of 0.2 μm is sufficient to remove all microbial cells and cell fragments (step VII). Afterwards, the corresponding filtrate contains the soluble target enzyme. Intracellular cellulases need a preceding extraction step (step VI). This is done by chemical cell lysis since mechanical procedures or sonification are not applicable for HT processing on a liquid handling robot platform. After the enzyme release by chemical cell lysis, the remaining cell fragments are removed by microfiltration as described earlier for secreted enzymes. It must be considered that the resulting filtrate contains all other soluble components from the cultivation step. But since this work focused on the expression of cellulases and not on their detailed characterization, these impurities are accepted due to the fact that all investigated enzyme candidates are affected in the same way.

The sample analysis aims for the activity measurement of expressed cellulases. Therefore, two methods for cellulase activity measurement were adapted for automated HTS. The colorimetric Azo-CMC test quantifies a blue dye which is released during cellulase driven CMC hydrolysis. According to the manufacturer’s instructions, this method is most effective for endoglucanases. Nevertheless, the applicability for cellobiohydrolases was proved within this work (data not shown). Alternatively, the reducing sugar assay PAHBAH can be used for cellulase quantification [29, 30]. Additionally, a third assay was applied for cellulase activity determination applying the fluorescent dye releasing substrate 4-MUC. This assay was not performed automatically yet, due to its fast and simple manual procedure. However, a full automation would be easy to realize with a sample amount of less than 48 wells.

The control of the robot system is based on a modular structure. Each processing step has its own routine. Depending on the demands of the experiment, all necessary steps can be combined. In this way, the flexible use of the system is ensured and routine programming is simplified. On the other hand, it might be disadvantageous that several steps cannot be operated in parallel, eventually causing increased processing times.

**Fig. 2:**
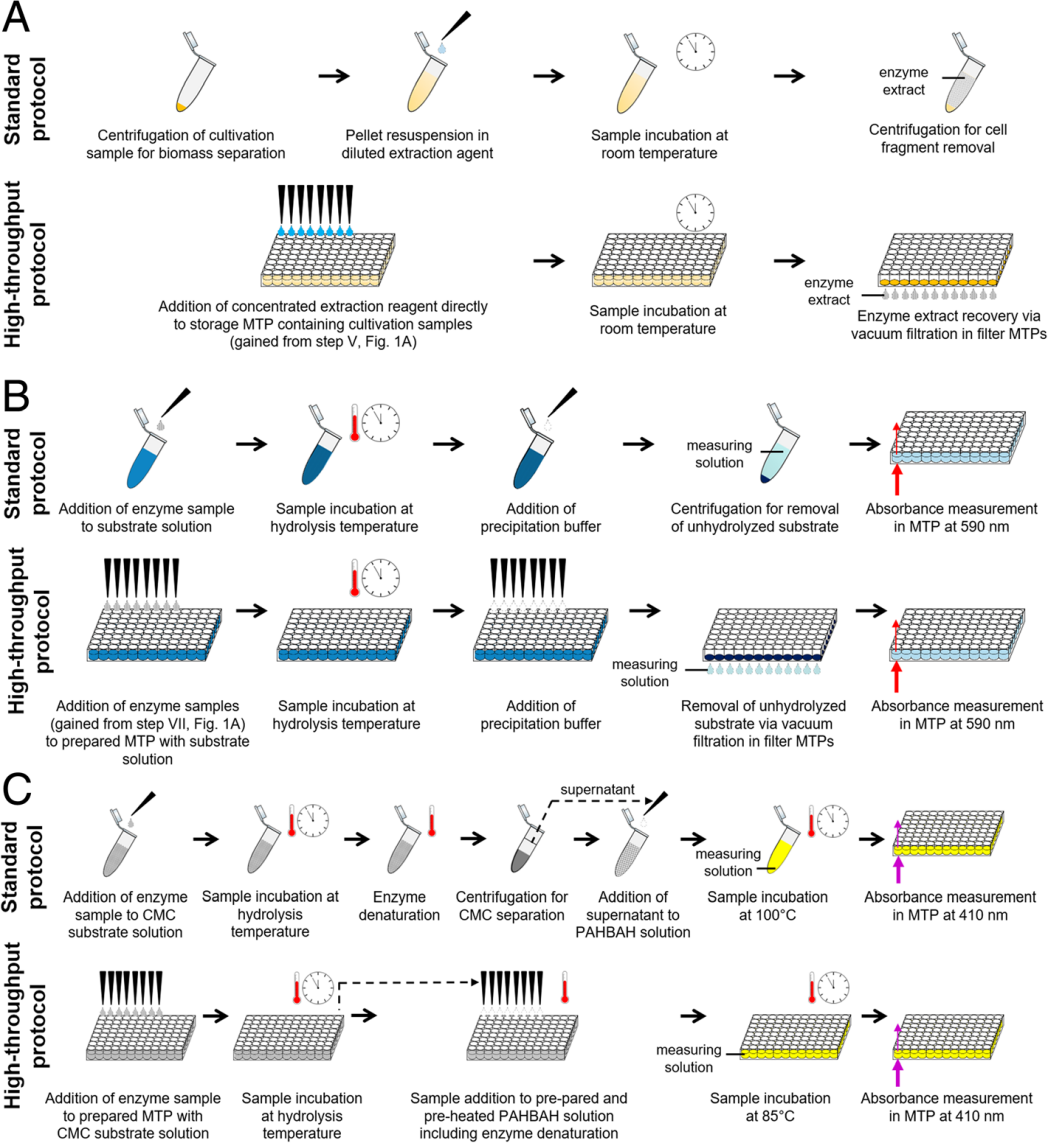
Schematic comparison of conventional protocols and modified protocols adapted for HT application in MTPs. **a** Extraction of recombinant enzymes/proteins from *E. coli* by chemical cell lysis applying BugBuster® reagent. **b** Colorimetric AzoCMC assay for cellulase activity measurement. **c** PAHBAH test for quantification of reducing sugars after CMC hydrolysis

Several process steps from Fig. 1b were not yet designed for a liquid handling robot platform, namely enzyme extraction (VI), biomass separation (VII) and the cellulase activity assays (VIII). Consequently, modifications were necessary for down-scaling and automation. Figure 2 compares schematically the standard lab protocols with the modified protocols for an application on the extended RoboLector system. In the following text, these modifications are described in detail.

### Recombinant protein extraction

For the extraction of recombinant proteins from *E. coli* cells, chemical cell lysis was chosen applying the BugBuster® reagent kit. In Fig. 2a the standard protocol according to the operator’s manual is compared with the modified HT protocol for automated handling. The conventional procedure applying 1.5 mL tubes started with a centrifugation step of cell suspension gained from a cultivation sample to separate biomass from the fermentation medium. After removal of the supernatant the cell pellet was re-suspended applying the extraction reagent. Therefore, the supplier is offering a 10fold concentrated solution which was appropriately diluted. After incubation at RT the mix was centrifuged again to remove solid components such as cell fragments. The resulting supernatant contained the target protein in soluble form. In contrary, the HT protocol was performed in 96well MTPs. Since centrifugation was not possible on the RoboLector platform, the respective steps had to be replaced. An alternative is microfiltration applying multiwell filter plates. In this way, whole cells and cell fragments can be sufficiently removed from liquid media. But as it turned out, complete re-suspension of cell pellets after the filtration was not possible due to a resistant filter cake. Consequently, the first two steps of the conventional protocol, namely biomass separation and re-suspension, were replaced by only one step. Therefore, the 10fold concentrated extraction reagent solution was not diluted but given directly to the cultivation sample including biomass and liquid medium. Nevertheless, the final reagent concentration before incubation was equal to the conventional protocol since the fermentation medium served as solvent. After sample incubation on a heater shaker (Fig. 1a, no. 6) at RT all solid components were removed by microfiltration applying 96well filter plates equipped with a membrane of 0.2 μm pore size on the vacuum filtration module (Fig. 1a, no. 7a). The resulting filtrate in the receiver plate contained the target protein in soluble form. To simplify the process and save material and time, all steps were performed in the same MTP. Consequently, in this case the filter plate served for sample storage, the extraction reaction and, finally, for cell fragment separation.

**Fig. 3:**
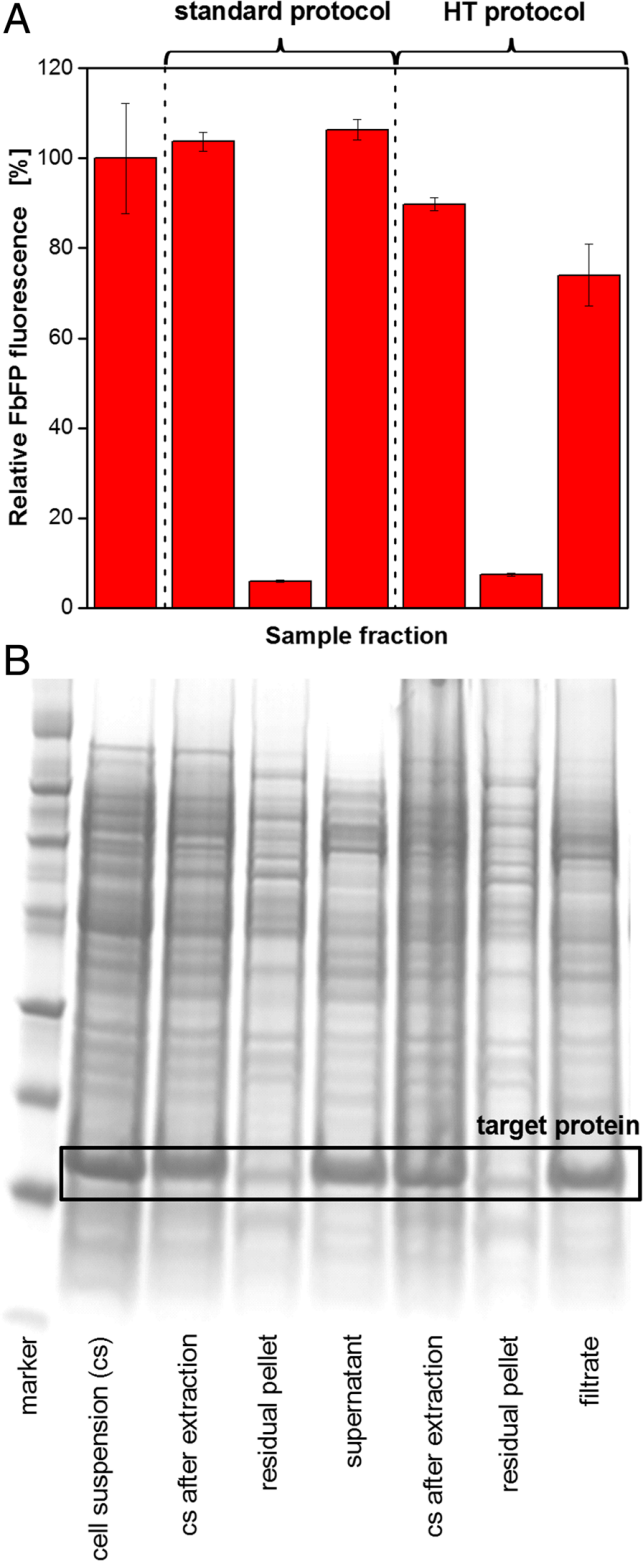
Extraction of the fluorescent model protein FbFP from *E. coli* BL21(DE3) pRhotHi-2 comparing the standard and HT protocol (according to Fig. 2a). **a** Fluorescence measurement of different fractions occurring during the extraction procedure applying the BioLector technique. *Error bars* indicate standard deviation of three simultaneously performed extraction experiments. **b** SDS-PAGE analysis of different fractions occurring during the extraction procedure. Target protein FbFP framed. Conditions: Cultivation in 10 mL OnEx auto-induction medium, 250 ml shake flask, *n* = 350 rpm, d_0_ = 50 mm, 37 °C. Extraction with BugBuster 10× Protein Extraction Reagent

To validate the modified HT protocol, samples from a cultivation of *E. coli* expressing the fluorescent protein FbFP were treated with both protocols and the extraction results were determined by fluorescence measurement and SDS-PAGE analysis (Fig. 3). For fluorescence measurement, the fluorescence intensity of the original cell suspension was used as a reference so that the first column in Fig. 3a shows a relative FbFP fluorescence of 100%. In the corresponding lane of the SDS-PAGE gel in Fig. 3b a prominent band (framed) can be seen, indicating the target protein. For the comparison of the standard and the HT protocol, three fractions which occur during the extraction process were analyzed: the cell suspension after the incubation with the extraction reagent, the pellet after cell fragment removal and the supernatant or filtrate, respectively, after cell fragment removal. For the standard protocol it can be seen that both, cell suspension after the extraction and supernatant after biomass removal, show relative fluorescence intensities around 100%. The SDS-PAGE gel confirms these measurements, showing strong bands for the target protein (framed) in both fractions. Almost no fluorescent target protein was found in the residual pellet. This means that the standard protocol works properly. The HT protocol shows similar results. The cell suspension after extraction gives a fluorescence intensity of 90%, the filtrate after cell fragment removal 74%, and the retained cell fragments a very low value of 7%. The SDS-PAGE gel is in good agreement with these values.

Even though, the measured values, especially those for the fraction containing the target protein, are lower for the HT protocol, the applicability for the RoboLector system could be shown since the majority of the target protein was found in the filtrate. It must be considered, that fluorescence measurement depends on many factors such as pH and ion concentrations [31]. After the extraction from the protecting cells, the FbFP was exposed to the respective environment. Since the medium composition slightly differed in the standard and HT protocol, the protein’s fluorescence might be affected leading to inaccurate results. The SDS-PAGE gel, on the other hand, shows very similar bands for the target protein for both protocols.

### Colorimetric Azo-CMC test

Assays applying dyed substrates are a convenient way to quantify enzymatic activity. For cellulases, Azo-CMC is a well-established substrate which releases a blue dye during hydrolysis. Subsequently, cellulase activity can be easily determined via absorbance measurement. However, this assay wasn’t yet applied for automated HTS. One reason for this might be the difficult handling with a pipetting robot. Figure 2b shows the manually performed standard protocol as well as the modifications of the assay procedure to adapt it for the application on the RoboLector platform. For comparison, starting point was the addition of an enzyme sample to the ready prepared and pre-heated Azo-CMC substrate solution. The standard protocol was performed in 5 mL tubes. For incubation, the filled tubes containing 0.5 mL preheated enzyme sample and 0.5 mL preheated substrate solution were shaken on a tube shaker for 10 min at 45 °C (hydrolysis temperature). Subsequently, the reaction was stopped by adding 2.5 mL precipitation buffer. To remove the precipitated non-hydrolyzed substrate, samples were centrifuged for 10 min at 18,000 g and 100 μL of supernatant (measuring solution) was transferred to a MTP for absorbance measurement. Thereby, it was critical to leave the unstable pellet unaffected since dispersed particles falsify the measurements. For the HT application, the assay was adapted to 96well DWPs. After addition of 200 μL enzyme samples to the DWP placed on the heater shaker containing 200 μL pre-heated Azo-CMC solution, the plate was incubated for 10 min at 45 °C at 900 rpm. By adding 1 mL precipitation buffer, the hydrolysis reaction was stopped. At this point, it was challenging to remove the precipitated substrate. On the one hand, the RoboLector is lacking a centrifuge. On the other hand, the reduction of the reaction volume due to the assay adapted to DWPs made it very difficult for the pipetting robot to remove a sufficient volume of supernatant without affecting the pellet. Considering the available hardware, a promising approach was microfiltration applying multiwell filter plates. It turned out that a membrane with a pore size of 0.2 μm was sufficient to retain the precipitated substrate particles (data not shown). After the filtration, 100 μL of the filtrate was transferred from the receiver plate to the final MTP for absorbance measurement in a MTP reader. It should be noticed that the MTP reader is not yet integrated into the RoboLector platform. Transport and measuring were done manually. Nonetheless, these steps could be automated in the foreseeable future.

**Fig. 4:**
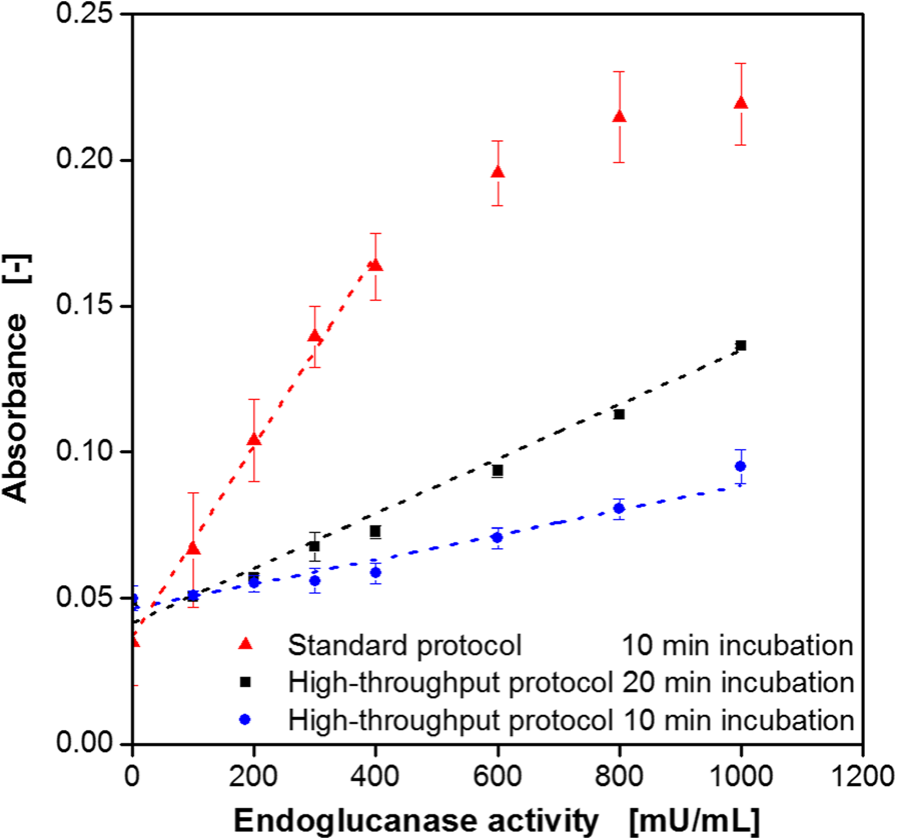
Calibration curves for the Azo-CMC cellulase activity assay comparing the standard protocol and HT protocol at varied incubation times (according to Fig. 2b). *Error bars* indicate standard deviation of three simultaneously performed assays. Assay conditions: incubation for 10 min in 5 mL tubes, V_L_ = 1 mL, *n* = 900 rpm, d_0_ = 3 mm (standard protocol). Incubation for 10/20 min in 96well DWP, V_L_ = 400 μL, *n* = 900 rpm, d_0_ = 3 mm (HT protocol). 0.2 g L^−1^ Azo-CMC in 0.1 M acetate buffer (pH = 4.8), endoglucanase (EGII) from *Trichoderma longibrachiatum*, 45 °C, absorbance measurement at 590 nm

To validate the modified HT protocol, calibration curves were determined with a commercially available endoglucanase. The relationship between enzyme activity and measured absorbance is shown in Fig. 4, comparing the manual standard protocol and the HT protocol at varied incubation times. It becomes obvious that the standard protocol (triangles) gives consequently higher values than the HT protocols. Better mixing conditions in the 5 mL tubes and, consequently, an improved substrate access for the enzyme might be the explanation. Nevertheless, a linear relationship is ensured for enzyme activities up to only 400 mU mL^−1^, before the curve runs into saturation. On the contrary, the HT protocol with 10 min incubation (circles) shows a linear trend up to 1000 mU mL^−1^. However, the narrow measuring range for the absorbance is rather disadvantageous. To face this problem, the incubation time was increased to 20 min (squares). In this way, the measuring range was more than doubled and measurements of high cellulase activities up to 1000 mU mL^−1^ were still possible. By looking at the respective standard deviations it must be stated that the accuracy of the HT protocols was much better compared to the manual standard protocol. This fact was ascribed to the filtration process which is much more efficient for the separation of the precipitated non-hydrolyzed Azo-CMC than centrifugation. Therefore, it was possible to adapt the colorimetric Azo-CMC assay for application on the RoboLector platform by modifying the assay procedure and optimizing the process parameters.

### PAHBAH reducing sugar assay

An alternative way to determine enzyme activity is the quantification of reaction products, which are reducing sugars for cellulases. The PAHBAH test is an already established assay to detect reducing ends. Even though applications in MTP applying a pipetting robot were reported earlier [32], a fully automated protocol has not been described so far. Figure 2c compares the standard manual test to the modified version for application on the RoboLector system. For comparison, the starting point was the addition of an enzyme sample to the ready prepared and pre-heated substrate solution. Exemplarily, CMC was chosen as substrate for cellulolytic enzymes. The standard protocol was performed in 1.5 mL tubes. For hydrolysis, the filled tubes containing 0.5 mL enzyme sample and 0.5 mL substrate solution were incubated on a tube shaker at 45 °C (hydrolysis temperature). By heating the samples up to 100 °C, enzymes were inactivated and the hydrolysis reaction stops. The following centrifugation causes the separation of long-chained CMC molecules in a viscous pellet from the less dense solution containing the short-chained sugar molecules in the supernatant. Subsequently, 75 μL supernatant was added to a tube containing 150 μL PAHBAH solution. After incubation at 100 °C for 10 min, samples were cooled to RT and 100 μL is transferred to a MTP for absorbance measurement. Since the assay quantifies reducing ends of released sugars, glucose was used for calibration. One glucose molecule possesses one reducing end that is detected by the assay. Therefore, released sugars are specified as glucose equivalents. For the HT application the assay had to be simplified and adapted to 96well DWPs. After addition of enzyme samples to the DWP placed on the heater shaker containing the pre-heated CMC solution, the plate was incubated for up to 5 h at hydrolysis temperature with shaking. Subsequent transferring of the hydrolysis samples to the DWP containing pre-heated PAHBAH reagent (at 85 °C) combined enzyme deactivation and the simultaneous start of the PAHBAH reaction. After incubating at 85 °C for 10 min, 100 μL samples were transferred to a MTP for absorbance measurement while cooling to RT. As already discussed for the AzoCMC assay, the transport to and measuring in the MTP reader was done manually. In contrast to the standard protocol, no separation of the long-chained CMC molecules was done after hydrolysis due to the unavailability of a centrifuge. In doing so, not only the reducing sugars in the supernatant were quantified but the increase of reducing ends during the hydrolysis of CMC. It must be considered that this only works out for low CMC concentrations since the sum of CMC reducing ends and those resulting from the hydrolysis must be within the linear range of the PAHBAH calibration.

**Fig. 5:**
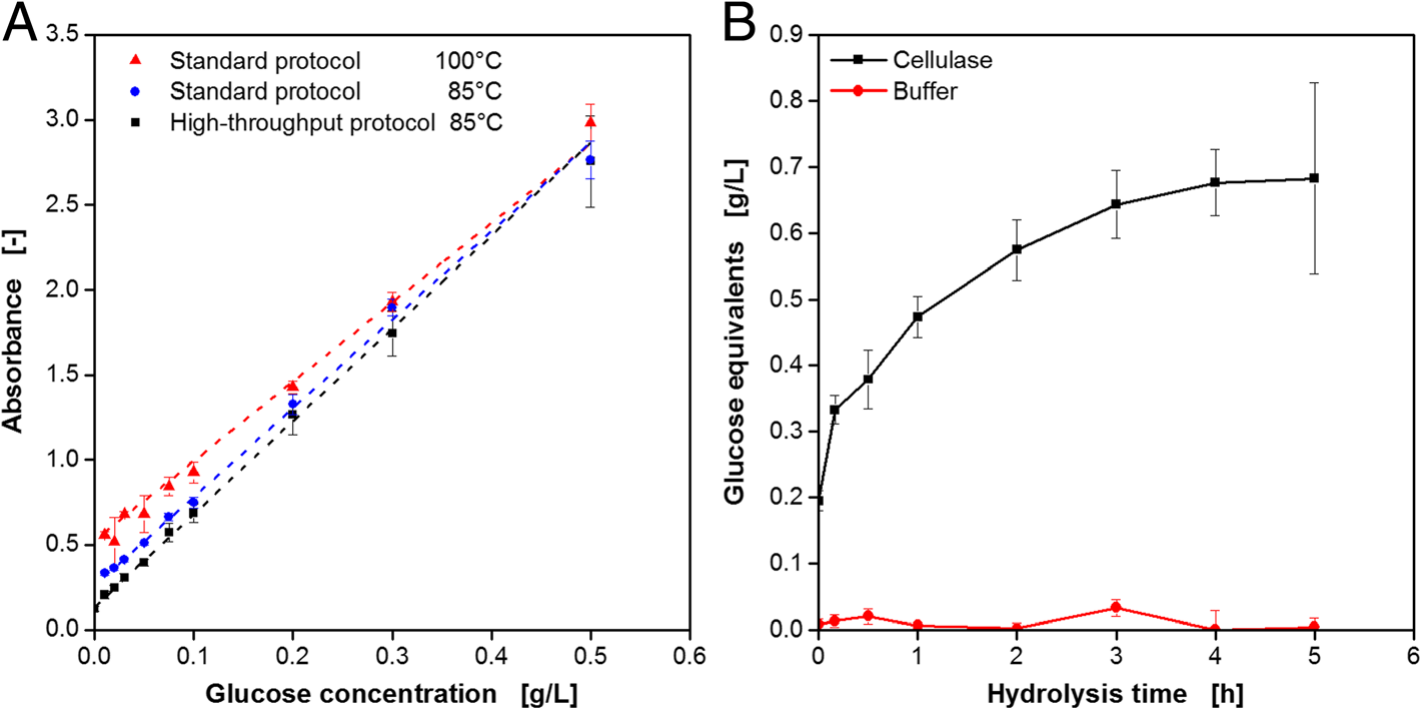
Adapting the reducing sugar assay PAHBAH for cellulose hydrolysis to automated HT conditions (according to Fig. 2c). **a** Calibration curves for the PAHBAH test comparing the standard protocol in tubes at varied incubation temperature and the HT protocol in MTPs. Conditions: Incubation in 1.5 ml tubes for 10 min, V_L_ = 225 μL, *n* = 900 rpm, d_0_ = 3 mm, 100/85 °C (standard protocol). Incubation in 96well DWP for 10 min, V_L_ = 225 μL, *n* = 900 rpm, 85 °C (HT protocol). Absorbance measurement at 410 nm. *Error bars* indicate standard deviation of three simultaneously performed assays. **b** Glucose equivalent formation during the hydrolysis of CMC incubated with 600 mU cellulase (EGII from *Trichoderma longibrachiatum*) or pure buffer solution measured via PAHBAH reducing sugar assay. *Error bars* indicate standard deviation of three simultaneously performed assays. Hydrolysis conditions: 96well DWP, V_L_ = 200 μL, 10 g L^−1^ CMC in 0.1 M acetate buffer, pH = 4.8, 45 °C, *n* = 900 rpm, d_0_ = 3 mm. PAHBAH assay conditions according to the HT protocol described for Fig. 5a

To validate the modified HT protocol, calibration curves with glucose were determined, comparing the standard protocol at varied temperatures with the HT protocol. Subsequently, the modified protocol was applied to follow CMC hydrolysis over time by measuring the reducing ends in terms of glucose equivalents. The results are depicted in Fig. 5. The calibration curves in Fig. 5a reveal very similar results for all three variants with a linear trend for glucose concentrations up to 0.5 g L^−1^. For the standard procedure, a temperature decrease during PAHBAH incubation from 100 °C (triangles) to 85 °C (circles) just caused slightly lower absorbance values at lower glucose concentrations. The temperature decrease became necessary since the maximum set temperature of the heater shakers is 110 °C. According to Eq. 2, this limits the incubation temperature in 96well DWP to 85 °C. Nevertheless, the PAHBAH test turned out to be reliable even at decreased temperatures. Mellitzer et al. [32] described a slight shift of the linear detection range to higher sugar concentration at lower temperatures. While it ranged from 0.01 to 1.4 mM (1.8 mg L^−1^–252.2 mg L^−1^) at 95 °C, it shifted to values of 0.12–2.45 mM (21.6 mg L^−1^–441.4 mg L^−1^) at 80 °C. These results were confirmed under the modified HT conditions since the measuring values are almost identical with those of the standard protocol at 85 °C. The corresponding calibration curve (squares) shows a very good linear trend for sugar concentrations up to 0.5 g L^−1^. It should be noted that for the calibration applying the HT protocol each sample contained 10 g L^−1^ CMC in addition to the glucose. For comparability with the standard protocol, the absorbance value for 10 g L^−1^ CMC and 0 g L^−1^ glucose was subtracted from each value.

Consequently, the PAHBAH test for reducing sugar detection could be successfully adapted to an automated HT handling on the RoboLector platform.

The novel tool was used to follow CMC hydrolysis by a fungal endoglucanase. Therefore, EGII from *T. longibrachiatum* was incubated with CMC solution in a 96well DWP on the heater shaker of the RoboLector. At pre-defined time points, samples were taken automatically and stored at 4 °C in a MTP on the cooling carrier. In parallel, hydrolysis experiments with pure buffer instead of enzyme solution were performed in the same plate as a reference. After the final samples were taken, all collected samples were analyzed via the PAHABH test. The results in Fig. 5b show the expected behavior. In the presence of the cellulase, the formation of glucose equivalents over time can be observed, with a steep slope in the beginning and saturation after 4–5 h. Without cellulase almost no sugars were detected over the whole time.

Subsequently, the RoboLector platform was used with the newly developed HT methods to characterize cellulase expression. Experiments were performed focusing on different aspects of cellulase expression and, consequently, requiring procedures of different complexity. In this way, different host organisms and expression systems were investigated and expression conditions were optimized. This was accompanied by a very high information content. The presented examples give an overview about the application possibilities and capabilities of the extended RoboLector system.

### Expression study of *K. lactis* for cellulase production

**Fig. 6:**
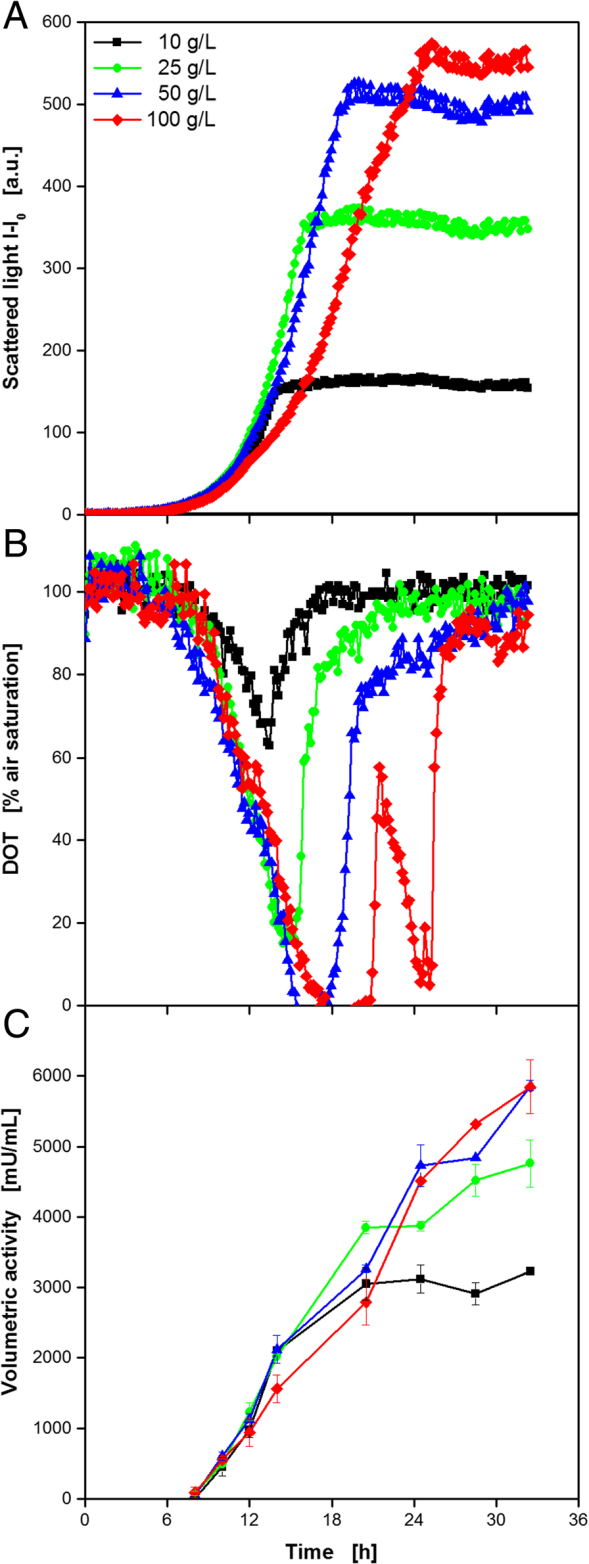
Expression study of *K. lactis* expressing recombinant endoglucanase cel5A from *Trichoderma reesei* at varied medium composition applying the RoboLector system. Cultivation and on-line monitoring of microbial growth (via scattered light, **a**) and DOT (**b**) at varied galactose concentrations. The data were derived from representative single experiments. They are in very good agreement with preliminary, non-automated characterization experiments focusing on growth kinetics and final product yield (data not shown). **c** Automated offline analysis of volumetric cellulase activity applying the AzoCMC assay. *Error bars* indicate standard deviation of three simultaneously performed assays. Cultivation conditions: 48well Flower plate, YP medium containing galactose as carbon source, V_L_ = 1 ml, *n* = 1500 rpm, d_0_ = 3 mm, 30 °C. Activity assay conditions: incubation in 96well DWP, 0.2 g L^−1^ Azo-CMC in 0.1 M acetate buffer (pH = 4.8), V_L_ = 400 μL, *n* = 900 rpm, d_0_ = 3 mm, 50 °C, absorbance measurement at 590 nm

For the first example, the behavior of the yeast *K. lactis* for the extracellular expression of recombinant endoglucanase cel5A from *T. reesei* was investigated. Therefore, the complex YP medium was automatically supplemented with different concentrations of galactose, which serves as substrate and at the same time as inducer. Subsequently, the media were transferred to a 48well Flower Plate and inoculated from the provided preculture. All these actions were performed via the pipetting robot. After sealing the plate and transporting it to the BioLector’s climate chamber, the cultivation was started. Up to now, automated plate sealing is not possible. But, it could be performed with the integration of the necessary equipment, which is commercially available, into the RoboLector system. Another task which requires manual intervention is the positioning of MTPs in the BioLector. The very tight holding mechanism cannot be handled with the robotic gripping tool. Hence, the development of a new mechanism controllable by the RoboLector software would be advantageous for further automation. Via optical on-line monitoring, microbial growth (scattered light) and DOT were determined over time, shown in Fig. 6a and b, respectively. It can be seen that all cultures started growing exponentially after a *lag* phase of 5–6 h, thereby showing almost identical growth rates until 12 h of cultivation. This is accompanied by an inverse decrease of the DOT signals. After 14 h, the culture with 10 g L^−1^ galactose reached the stationary phase, whereas it took 16 h and 19 h for the cultures with 25 g L^−1^ and 50 g L^−1^ galactose, respectively. The simultaneous increase of the DOT signal indicates the complete consumption of the primary substrate galactose. The three curves show no distinct signs of limitation. This is confirmed by the DOT signals which show minimum values of 63 and 16% for 10 and 25 g L^−1^ galactose, and only a short period of oxygen limitation for 50 g L^−1^. Also inhibitions, e.g. caused by osmotic stress or recombinant protein productions, seem not to be apparent. Contrary to that, the culture with 100 g L^−1^ galactose showed a first decrease in the growth rate after 12 h. The reason for this is not yet clear. Oxygen limitation can be excluded since the DOT at this time was still higher than 50%. Nevertheless, a short interruption in the DOT decrease could be observed at this time, too. In the following, microbial growth slightly recovered before becoming linear after 17.5 h due to an oxygen limitation. After 21 h, the DOT signal started increasing before another drop at 22 h accompanied by a further increase of the biomass signal, albeit with decreased growth rate. *K. lactis* is known to produce ethanol under oxygen limitation or as a product of overflow metabolism. Consequently, the second decrease of the DOT signal is due to the utilization of ethanol which was formed from galactose in the earlier stages of the cultivation. This phenomenon was described before [33]. After 25 h, the culture entered the stationary phase and the DOT recovered again.

For the quantification of cellulase production, samples were taken automatically, starting after 8 h of cultivation when the cultures had already entered the exponential growth phase. For sampling, whole wells of the cultivation plate in the BioLector were sacrificed and the cell suspension was transferred to the storage plate on the cooling carrier. In this case, a multiwell plate for microfiltration served for storage at 4 °C. After the final samples were taken, the filter plate with the samples was transported to the vacuum station, filtration was done, and the cell-free permeate was collected in a receiver DWP for subsequent analysis via Azo-CMC assay. Since the enzyme is secreted from the host cells, no further extraction step was necessary. The results in Fig. 6c show that all cultures had a strongly growth related cellulase production behavior. With the beginning of exponential growth, cellulase activity started increasing in the same way, reaching higher final activity values with higher initial galactose concentrations. However, an increase to 100 g L^−1^ galactose didn’t result in further improvement. Interestingly, cellulase production did not stop with biomass formation, but it continued until the DOT signal was fully recovered. Consequently, *K. lactis* had the ability for recombinant protein expression, even though the inducing component in the medium was depleted. Earlier reported carbon storage mechanisms might be the explanation for this phenomenon [34].

The results enable a comparison of biomass and product formation under consideration of the used substrate (data shown in Additional file 1: Table S1). The respective yields are an important parameter which influences how to run an industrial process. Therefore, the RoboLector system with its novel capabilities can deliver a good data base for making decisions.

### Auto-induction medium optimization for cellulase-expressing *E. coli*

**Fig. 7:**
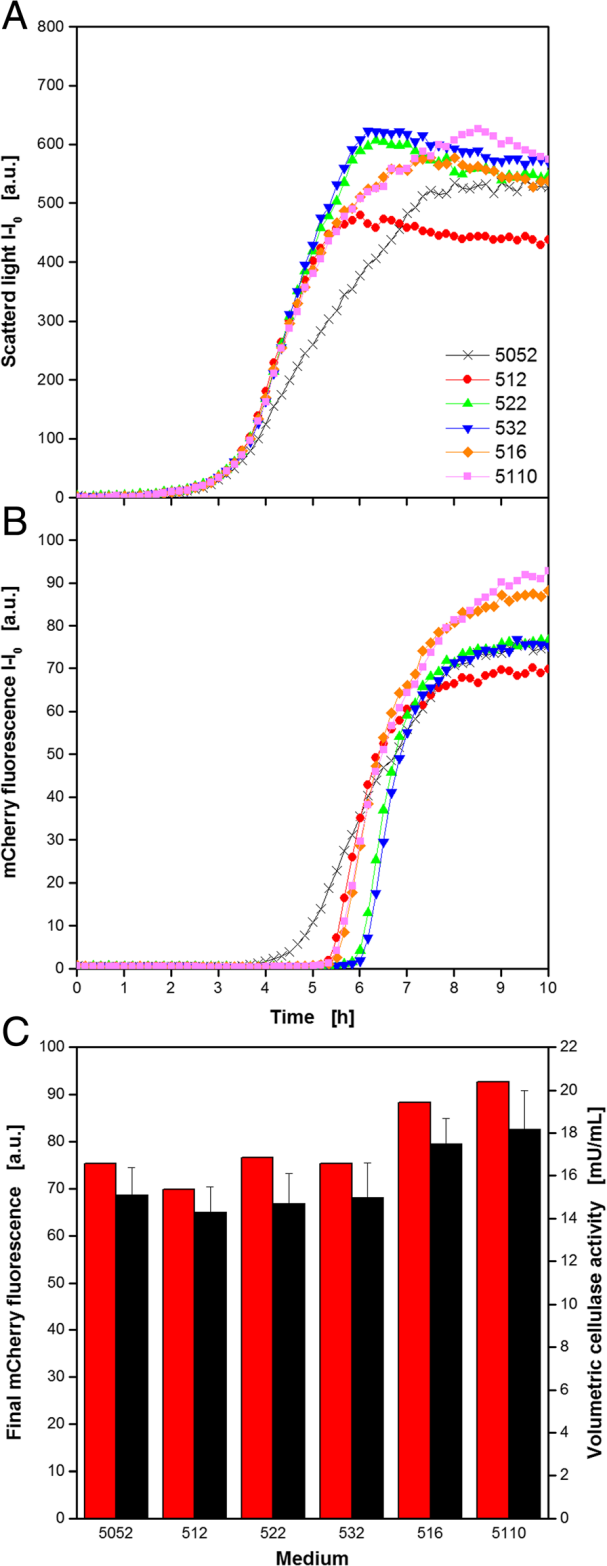
Auto-induction medium variation for *E. coli* expressing recombinant extremophile endoglucanase cel8H from *Halomonas sp.* tagged with fluorescent mCherry applying the RoboLector system. Cultivation and on-line monitoring of microbial growth (via scattered light, **a**) and recombinant cellulase expression (via mCherry fluorescence, **b**) for different auto-induction medium compositions (see Table 2). **c** Final mCherry fluorescence and volumetric cellulase activity applying the PAHBAH reducing sugar assay for different auto-induction medium compositions. *Error bars* indicate standard deviation of three simultaneously performed assays. Cultivation conditions: 96well MTP, TB medium with glycerol (5 g L^−1^) and varied glucose (0.5–3 g L^−1^) and lactose (2–10 g L^−1^) concentrations, V_L_ = 200 μl, *n* = 1000 rpm, d_0_ = 3 mm, 37 °C. Hydrolysis conditions: incubation in 96well DWP for 60 min, V_L_ = 200 μL, 10 g L^−1^ CMC in 0.1 M acetate buffer, pH = 5, 45 °C, *n* = 900 rpm, d_0_ = 3 mm. PAHABH assay conditions: incubation in 96well DWP for 10 min, V_L_ = 225 μL, *n* = 900 rpm, d_0_ = 3 mm, 85 °C, absorbance measurement at 410 nm

In a second exemplary study, the recombinant expression of the highly salt tolerant and thermostable endoglucanase cel8H from *Halomonas sp66-4* in *E. coli* was attempted to be improved by optimizing the carbon source composition of the applied auto-induction medium. Besides with the original carbon source glycerol, the complex TB medium was enriched with different amounts of glucose and lactose for auto-induction. This approach works as follows: glucose is the preferred carbon source and represses recombinant protein expression to ensure undisturbed initial growth. After glucose depletion lactose is taken up and acts as the inducer of the expression system. Glycerol is an additional energy source. It is consumed in parallel to lactose [33, 35, 36]. It was attempted to vary the time point of induction and the inducer concentration in order to influence the product formation [18, 36]. For a convenient quantification of the cellulase formation, the target protein was linked to a fluorescent mCherry reporter tag. The experiment started with the fully automated composition of the medium variants from a carbon source free TB medium and stock solutions for glucose, lactose, and glycerol directly in a 48well Flower Plate for cultivation. Subsequently, all wells were inoculated from the provided preculture. After sealing the plate and its transport to the BioLector, the cultivation was started. Via optical on-line monitoring, microbial growth (scattered light) and cellulase production (mCherry fluorescence) were determined over time. The results for six exemplary wells (out of 48) are shown in Fig. 7a and b. The legend for the code numbers is given in Table 2. It can be seen that all cultures start growing exponentially after a *lag* phase of 1.5 h, thereby showing identical growth rates until 3.5 h of cultivation. At this time, the growth in medium 5052, became slower compared to the other cultures. This is not surprising, since 5052 has the lowest glucose concentration which leads to the earliest time of lactose uptake and, consequently, to the induction of the recombinant protein expression. It is known that this can cause a metabolic burden to the host organism and, hence, inhibiting its growth [36]. Higher glucose concentrations allow a longer undisturbed growth and, therefore, shift the inhibition phase to a later time. For of 1 g L^−1^ (see 512, 516, and 5110) this was after approx. 5 h, whereas no clear growth inhibition occured with 2 and 3 g L^−1^ glucose (see 522 and 532). Comparing the curves for the media 516 and 5110, the effect of induction by lactose on growth becomes visible. Where 6 g L^−1^ lactose caused inhibition until 7.5 h, it took until 8.5 h with 10 g L^−1^ lactose. The final scattered light values differ among the investigated conditions as a result of several factors, e.g. the varied total carbon source amounts, growth inhibition, and varying product formation. It was reported before, that the red mCherry fluorescence affects the scattered light measurement at 620 nm which means that higher fluorescence intensities cause higher scattered light values [37]. When interpreting the BioLector signals, this should be taken into consideration. Consequently, the low final biomass signal for medium 512 is most likely a result of low recombinant protein expression (comp. Fig. 7b).

In accordance with the biomass formation, the cellulase production behavior differed among the media. The first medium showing an increase of the fluorescence signal after 3.5 h was the standard medium 5052, due to lowest glucose concentration. As expected, all media with 1 g L^−1^ glucose (512, 516, 5110) followed simultaneously after approx. 5 h, before those with higher concentrations (522, 532) started product formation after 6 h. As observed for microbial growth, no differences occurred for 2 and 3 g L^−1^ glucose. Compared to the rapid increase of all other media, the expression in the standard medium 5052 was rather slow. This can be explained by less biomass at the time of induction and the following stronger growth inhibition. Consequently, less producing host cells were available over the whole cultivation time. Nevertheless, the final product concentration was comparable to those media with 2 and 3 g L^−1^ glucose and even slightly higher than for 1 g L^−1^. It has to be mentioned, that lactose was used as substrate, too. As a consequence, the same amount of lactose is utilized faster if more cells are present. This leads to an earlier depletion of the inducer lactose and, hence, to an earlier stop of cellulase expression. This assumption is proved by the curves for 516 and 5110, where additional lactose caused the highest measured target protein formation.

To countercheck the fluorescence values, samples were taken at the end of the cultivation and enzyme activity was determined by CMC hydrolysis and subsequent PAHBAH test applying the HT protocol with the RoboLector system. Therefore, the cell suspension was directly transferred to a 96well microfiltration plate. Since the target protein was expressed intracellularly it was extracted from the *E. coli* cells applying the HT protein extraction protocol described before (Fig. 2a). Subsequently, filtrate samples were used for HT CMC hydrolysis and reducing sugars were quantified by PAHBAH reaction. With the measured values, the volumetric cellulase activity was calculated. One unit was defined as 1 μmol of glucose equivalents produced per minute of reaction time. In Fig. 7c, the final mCherry fluorescence intensities are compared to the final volumetric enzyme activities. Signals went hand in hand since higher fluorescence values are accompanied by higher activities and vice versa. The best result was found for medium 5110, showing approx. 20% more product formation than the standard medium 5052. Additionally, the data showed that the specific activity of the cellulase did not change with the varied medium composition for enzyme production.

This example showed how the RoboLector system can conveniently be used to optimize a cellulase production process. The combination of fully automated medium composition, on-line monitoring of biomass and product formation, and final activity measurement allows fast process characterization and is a good data base for further process development. For this goal, the fluorescent tag on the enzyme is a valuable tool to gain kinetic information. Furthermore, the very sensitive PAHBAH test even allows the detection of rather low activities of the highly salt tolerant enzyme cel8H. Such enzymes gain more and more attention since bioprocesses often run under harsh conditions. Cel8H is still active at very high salt loads and, hence, an interesting candidate for future research [38]. Among other measures, elevated expression levels as achieved with the medium optimization can help to improve the feasibility of a potential biomass conversion process.

### Advanced primary clone bank screening

**Fig. 8:**
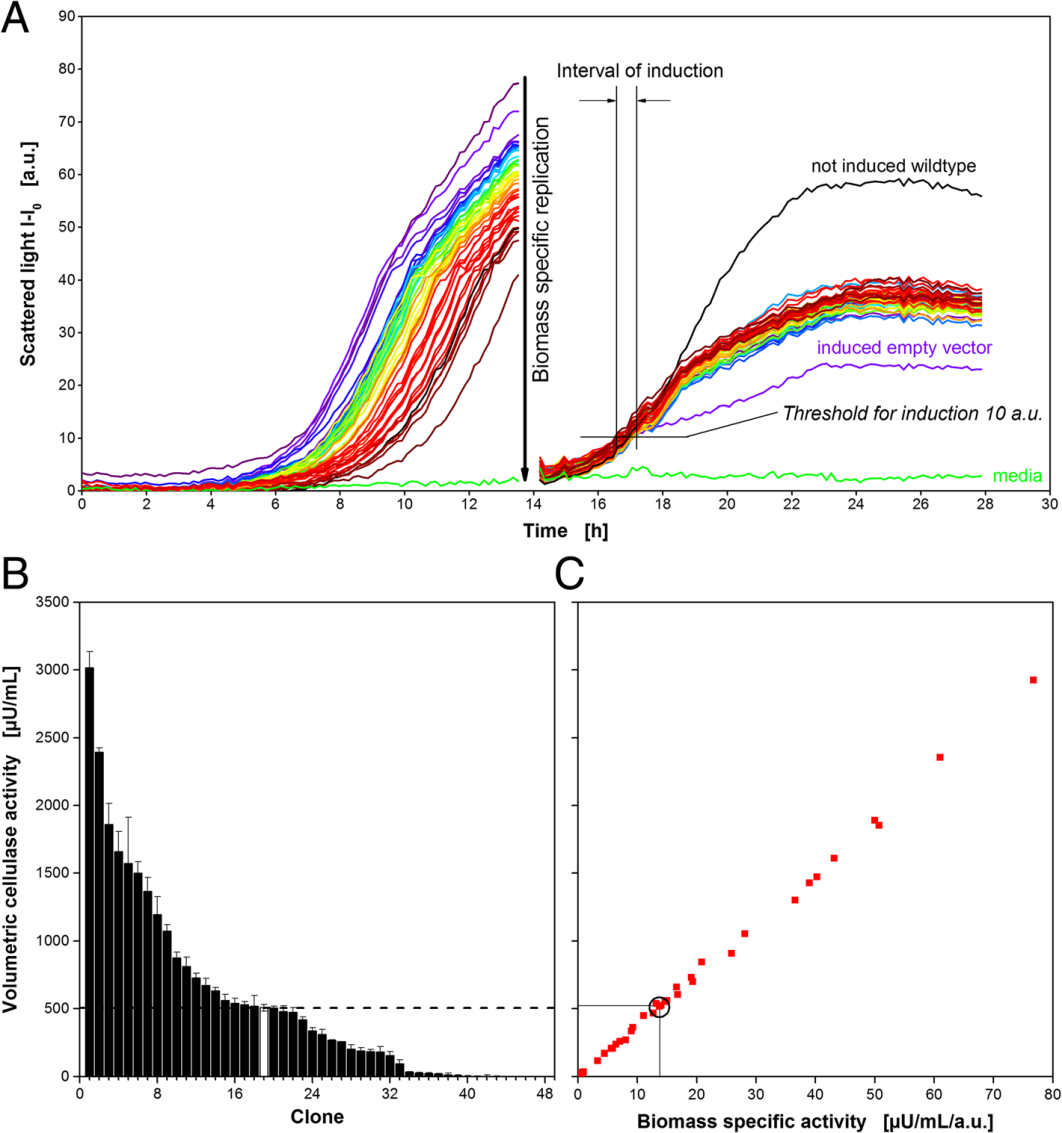
Primary screening of an *E. coli* random mutagenesis clone library expressing variants of endoglucanase celA2 applying the RoboLector system. **a** On-line biomass signal (monitored via scattered light) for a two-step cultivation of 46 *E. coli* clones consisting of one not induced pre-cultivation and an induced main cultivation. Additionally, a not induced wildtype and plain media served as references. The main cultivation was automatically inoculated from the previous cultivation step. **b** Volumetric cellulase activity (via 4-MUC assay) of the supernatant after cellulase extraction from *E. coli* cells including standard deviations of three independent main cultivations. Reference activity of the clone expressing the original enzyme indicated by *dashed line* and *white bar*. Not induced wildtype and media are depicted as clone no. 47 and 48. **c**) Parity plot comparing volumetric and biomass-specific cellulase activities of the supernatants after cellulase extraction from *E. coli* cells of the main cultivation. For calculation of biomass specific activities, the final scattered light values of the main cultivation were used. Wildtype clone expressing the original enzyme indicated by *circle*. Cultivation conditions: 96well MTP, V_L_ = 150 μL, *n* = 995 rpm, d_0_ = 3 mm, pre-cultivation and main cultivation in Wilms-MOPS medium containing 15 g L^−1^ glucose, cellulase expression automatically induced by adding 0.1 mM IPTG after reaching the scattered light threshold of 10 a.u.. Activity assay conditions: incubation in 96well MTP, 0.05 mM 4-MUC solution in potassium phosphate buffer (pH = 6.5), V_L_ = 150 μL, 30 °C, fluorescence measurement at 365/455 nm

Figure 8 depicts the results of the most complex example performed within this study. The data was generated during a primary clone bank screening for the expression of 46 variants of endoglucanase celA2 in *E. coli*. The enzyme variants were created by directed evolution applying a triple site-saturation mutagenesis [24, 39, 40]. This screening protocol consisted of a two-step cultivation including one pre-cultivation and a main cultivation with automated biomass specific induction. To enable a complete automated cultivation, the first six columns of the plate were used for the preculture and the last six columns for the main culture. This avoided the manual transport of MTPs out of the BioLector. For reference, one well in each step of the cultivation was filled with plain medium. Moreover, the wildtype was present two times in both cultivation steps. One served as a non-induced reference.

At the end of the cultivation, the cultures were harvested and used for cell lysis. The already published method to determine celA2 activity in MTP format is the 4-MUC assay [24]. The volumetric activity was calculated from a kinetic measurement of the increase in released fluorescent 4-MU. Therefore, a quick measurement of all micro-wells was necessary. This was not possible within the BioLector since it takes about 4 min to measure 96 wells. Therefore, a manual offline activity assay using a Synergy 4 Microplate reader was chosen for the evaluation of this clone library. But it’s important to consider, that the final assay only measures the volumetric activity. As described in Eq. 3, a change in volumetric activity can be caused by a difference in the enzymatic specific activity (term 1), by a difference in the biomass specific activity (term 2), or by a difference in the biomass concentration (term 3).

3 $$ \frac{\mathrm{U}}{\mathrm{mL}}=\frac{\mathrm{U}}{{\mathrm{g}}_{\mathrm{enzyme}}}\cdot \frac{{\mathrm{g}}_{\mathrm{enzyme}}}{{\mathrm{g}}_{\mathrm{biomass}}}\cdot \frac{{\mathrm{g}}_{\mathrm{biomass}}}{\mathrm{mL}} $$

Since the volumetric activity is dependent on these three parameters and only the last term describing the biomass concentration is known from scattered light measurements, it is in most cases not possible to distinguish whether the differences are caused by the first or second term. Even a concurrent effect of all three terms cannot be excluded. Dependent on the purpose of a library screening, a subsequent enzyme quantification would be necessary to yield the enzyme specific activities. HT affinity chromatography in MTP-format are commercially available for this task, however this leads in most cases to an undesired increase of investment. An easy solution for this problem would be to label the target proteins with fluorescent tags. Term 2 and 3 from Eq. 3 would then be known and the enzymatic specific activity could be calculated. However, tags with high molecular weight may stress the host cell metabolism and thus change the target protein expression efficiency or even the enzyme activity. Using smaller tags like tryptophan tags [41] or splitted tags such as the Split-GFP technology [42] could be the method of choice.

The scattered light curves of all clones in Fig. 8a show different lag phases within the preculture. A time shift of up to 4 h within the exponential growth phase was observed when comparing the variants. This is most likely due to fluctuations in the initial cell concentration which are commonly present in cryo cultures of clone libraries. These differences are then transferred into the culture plate. Moreover, the replication tool which was used to transfer the cells from the cryo plate to the culture plate plays an important role, as described elsewhere [23]. The asynchronous growth was eliminated after operating the automated biomass specific normalization step shortly after 13 h. Nevertheless, an automated biomass specific induction was followed which ensures identical induction conditions. As soon as the scattered light exceeded the preset threshold of 10 a.u. the robot induces these specific wells with 10 μL of IPTG, resulting in a final concentration of 0.1 mM. Interestingly, all variants behave the same way after induction and end up with the same biomass concentration at the end of the cultivation. Consequently, similar metabolic burden and similar protein production can be assumed. Integration of this assumption into Eq. 3 lead to a direct proportionality of volumetric activity and enzyme specific activity. Thus, an elaborate quantification of the celA2 protein content is not necessary.

After about 14 h of main culture, the cell suspensions were harvested and cell pellets were lysed using BugBuster. The volumetric activity of received crude extract was determined by the fluorescence-based 4-MUC assay. The final clone ranking is depicted in Fig. 8b. Mean values of cellulase activity and error bars coming from three independently conducted HTS are shown. This includes the experiment depicted in Fig. 8a and two more experiments whereof no scattered light curves are shown here. The volumetric activity of the induced wildtype is illustrated as white bar. A six times higher activity was obtained by the best variant when compared to the wildtype. A very small relative standard deviation is observed (wildtype, 5.1%; best clone, 4.0%). This confirms a very high reproducibility of the screening method using the automated preculture synchronization and the biomass specific induction. Furthermore, it gives a maximum of standardization accompanied by an on-line monitoring signal to control it. Considering the final scattered light values of the cultures, biomass specific cellulase activities can be determined. Figure 8c describes the dependency of volumetric activity and biomass specific enzyme activity. Since the biomass concentration was similar for all clones, a completely linear trend was observed.

## Conclusion

The RoboLector platform was successfully extended by a cooling carrier, a MTP shaker for heat treatment and a vacuum filtration module. This enables the robot to perform a cellulase screening experiment consisting of upstream, downstream and analysis steps. Some downstream and analysis protocols were adapted from manual handling to a HT protocol suitable for automated handling. This comprises the enzyme extraction step, the Azo-CMC and the PAHBAH cellulase activity assays.

The established methods were successfully applied to investigate the expression of cellulase cel5a in *K. lactis* and to optimize the expression of the endoglucanase cel8H tagged with mCherry in *E. coli*. The results showed how the RoboLector system can conveniently be used to optimize media compositions and analyze the outcome without manual intervention.

The automated HTS of the *E. coli* celA2 library showed the advantage of a biomass specific replication step, to achieve synchronized cultures. A highly parallel biomass growth could be achieved and via biomass specific induction a comparable expression of target protein was secured. A clone ranking revealed a six times higher activity of the best variant, compared to the wildtype.

It was demonstrated that a large variety of methods could successfully be automated on the extended RoboLector platform. The device allows a strongly increased information content compared to screening systems reported so far. The results confirm the feasibility of a reliable and automated HTS on the robotic platform. Up to now, only a small clone library consisting of 46 different variants was screened. The future strategy will be the automation of the whole cellulase screening system with emphasis on the linkages between the already described automated modules and finally screen a large clone library. A comparison of the manual and automated screening is certainly of great interest and will be part of upcoming experiments.

## Additional file

Additional file 1: Table S1. Relative yield coefficients for the expression study with *K. lactis* expressing recombinant endoglucanase cel5A from *Trichoderma reesei* with varied galactose concentration. The data were derived from representative single experiments. Y_X/S_= Biomass yield on substrate, Y_P/S_= Product yield on substrate, STY= Space time yield. (DOCX 13.2 kb)

## Abbreviations

4-MUC: 4-methylumbelliferyl-β-D-cellobioside
a.u.: Arbitrary unit
Azo-CMC: Carboxymethyl cellulose dyed with Remazolbrilliant Blue
cel5A: Enduglucanase from *Trichoderma reesei*
cel8H-mCherry: Enduglucanase from *Halomonas sp.* tagged with the fluorescent protein mCherry
celA2: Cellulase originally isolated from a metagenome library
CMC: Carboxymethyl cellulose
DNS: 3,5-dinitrosalycilic acid
DOT: Dissolved oxygen tension
DWP: Deep well plate
*E. coli*: *Escherichia coli*
EG: Endoglucanase
Em: Emission
Ex: Excitation
FbFP: Flavin mononucleotide (FMN)-based fluorescent protein
HT: High-throughput
HTS: High-throughput screening
I: Intensity
I_0_: Initial light intensity
IPTG: Isopropyl β-d-1-thiogalactopyranoside
*K. lactis*: *Klyveromyces lactis*
MOPS: 3-(N-morpholino)-propanesulfonic acid
MTP: Microtiter plate
PAHBAH: *Para-*hydroxybenzoic acid hydrazide
rpm: Round per minute
RT: Room temperature
SDS-PAGE: Sodium dodecyl sulfate polyacrylamide gel electrophoresis
STY: Space time yield
T: Temperature
t_1;…;n_: Sampling time
TB: Terrific broth
U: Enzyme unit
YP: Yeast extract peptone
Y_P/S_: Product yield from substrate
Y_X/S_: Biomass yield from substrate
λ_em_: Emission wavelength
λ_ex_: Excitation wavelength
