# Supplementary material for: Cellulolytic RoboLector – towards an automated high-throughput screening platform for recombinant cellulase expression
Source: J Biol Eng. 2017 Jan 7;11:1. doi: 10.1186/s13036-016-0043-2 (PMC5219752; doi:10.1186/s13036-016-0043-2)
Supplement: Additional file 1: Table S1. — Relative yield coefficients for the expression study with K. lactis expressing recombinant endoglucanase cel5A from Trichoderma reesei with varied galactose concentration. The data were derived from representative single experiments. YX/S= Biomass yield on substrate, YP/S= Product yield on substrate, STY= Space time yield. (DOCX 13.2 kb) [file 13036_2016_43_MOESM1_ESM.docx]

# Additional File 1

**Table S1** – Relative yield coefficients for the expression study with K. lactis expressing recombinant endoglucanase cel5A from Trichoderma reesei with varied galactose concentration. Taking the culture with 10 g L^-1^ galactose as a reference. The data were derived from representative single experiments. Y_X/S_ = Biomass yield on substrate, Y_P/S_= Product yield on substrate, STY= Space time yield.

| Galactose conc.  [g L^-1^] | Relative Y_X/S_  [%] | Relative Y_P/S_  [%] | Relative max. product STY  [%] |
| --- | --- | --- | --- |
| 10 | 100 | 100 | 100 (after 20 h) |
| 25 | 89 | 59 | 124 (after 20 h) |
| 50 | 63 | 36 | 152 (after 20 h) |
| 100 | 34 | 18 | 145 (after 20 h) |

It can be seen that an increase of the substrate concentration led to a decrease of the yields for biomass and product formation. Even though, the maximum yield for cellulase production was found for all cultures after 20 h, it was highest with 50 g L^-1^ galactose. Consequently, for a later application of the *K. lactis* expression system for endoglucanase production, priorities need to be defined in order to choose the optimal substrate concentration, thereby considering the factors time, substrate costs, and desired product concentration.
